# Myosin filament 3D structure in mammalian cardiac muscle^☆^

**DOI:** 10.1016/j.jsb.2008.03.011

**Published:** 2008-08

**Authors:** Hind A. AL-Khayat, Edward P. Morris, Robert W. Kensler, John M. Squire

**Affiliations:** a Institute of Biomedical Engineering, Imperial College London, Bessemer Building, London SW7 2AZ, UK; b Institute of Cancer Research, 237 Fulham Road, Chester Beatty Laboratories, London SW3 6JB, UK; c Department of Anatomy, University of Puerto Rico Medical School, San Juan 00936-5067, Puerto Rico; d Muscle Contraction Group, Department of Physiology and Pharmacology, University of Bristol, Bristol BS8 1TD, UK

**Keywords:** Myosin filaments, Single particle analysis, 3D reconstruction, Rabbit cardiac muscle, MyBP-C, Mammalian heart muscle

## Abstract

A number of cardiac myopathies (e.g. familial hypertrophic cardiomyopathy and dilated cardiomyopathy) are linked to mutations in cardiac muscle myosin filament proteins, including myosin and myosin binding protein C (MyBP-C). To understand the myopathies it is necessary to know the normal 3D structure of these filaments. We have carried out 3D single particle analysis of electron micrograph images of negatively stained isolated myosin filaments from rabbit cardiac muscle. Single filament images were aligned and divided into segments about 2 × 430 Å long, each of which was treated as an independent ‘particle’. The resulting 40 Å resolution 3D reconstruction showed both axial and azimuthal (no radial) myosin head perturbations within the 430 Å repeat, with successive crown rotations of approximately 60°, 60° and 0°, rather than the regular 40° for an unperturbed helix. However, it is shown that the projecting density peaks appear to start at low radius from origins closer to those expected for an unperturbed helical filament, and that the azimuthal perturbation especially increases with radius. The head arrangements in rabbit cardiac myosin filaments are very similar to those in fish skeletal muscle myosin filaments, suggesting a possible general structural theme for myosin filaments in all vertebrate striated muscles (skeletal and cardiac).

## 1 Introduction

Myosin filaments (also known as thick filaments), which interact with actin filaments in muscle to produce force and movement, are assemblies of myosin molecules and accessory proteins. Much is known about their 3D structure, but high resolution information is hard to obtain. Mutations in cardiac muscle myosin and its associated proteins (e.g. C-protein; myosin binding protein C (MyBP-C)) are known to be associated with a number of myopathies (e.g. familial hypertrophic cardiomyopathy and dilated cardiomyopathy) (Watkins et al., 1992, 1995; Seidman and Seidman, 2001; Tajsharghi et al., 2003). To understand how heart muscle normally works and how the known mutations affect contractility it is essential to understand the structure and properties of normal mammalian cardiac myosin filaments.

Myosin molecules comprise two heavy chains and four light chains. Parts of the heavy chains twist together to form a 1500-Å long coiled-coil α-helical rod-shaped tail domain on one end of which are two elongated globular myosin heads (crossbridges) formed by the rest of the heavy chains together with the light chains. The way in which myosin molecules and accessory proteins associate to form a myosin filament can vary depending on the type of muscle. Generally the tails pack together, often in a helical arrangement, to form the filament backbone from which the heads project. The heads are ATPases that interact with actin filaments to produce force and movement (Huxley, 1963). In vertebrate striated muscle the myosin molecules pack together into bipolar myosin filaments with the rods forming a roughly cylindrical filament backbone and the myosin heads arranged in a quasi-helical array on the filament surface. Halfway along each filament, where the rod packing is anti-parallel, there is a head-free region known as the bare-zone or M-region (Huxley, 1963; Sjostrom and Squire, 1977a, 1977b). The first myosin heads in each half A-band are at the edges of the M-region. The backbone also has on its surface additional non-myosin protein components including C-protein (MyBP-C; Offer et al., 1973); or possibly its analogue X-protein (Bennett et al., 1986) and the A-band part of titin (Labeit and Kolmerer, 1995; Trinick, 1996).

The head arrangement on vertebrate striated muscle myosin filaments approximates to a 3-stranded right-handed helix where each strand possesses nine subunits (pairs of myosin heads) per turn and a pitch of 1287 Å. Since the filament is 3-stranded, the axial repeat is reduced from 1287 Å to 430 Å. The pairs of heads from three myosin molecules project from the backbone at regular intervals to form a so-called ‘crown’ of heads, with successive crowns separated axially by roughly 143 Å (Squire, 1972). The angle between each of the three head pairs on one crown is 120°. Each 430 Å repeat contains three such crowns (Squire, 1972, 1973, 1974; Harford and Squire, 1986; Stewart and Kensler, 1986; Cantino and Squire, 1986; Kensler and Stewart, 1989). Vertebrate striated muscle myosin filaments are characterised by a systematic departure from perfect helical symmetry, known as a perturbation. This was originally identified by the presence of ‘forbidden’ meridional reflections (i.e. X-ray meridional reflections seen at orders of the 430 Å repeat other than multiples of 3) in the X-ray diffraction patterns from relaxed vertebrate skeletal muscles (Huxley and Brown, 1967; Squire et al., 1982; Harford and Squire, 1986).

Electron microscopic analysis of vertebrate striated muscle myosin filaments has included 2D analysis of images of negatively stained myosin filaments from fish, frog, chicken, and rabbit skeletal muscles (Kensler and Stewart, 1983, 1989, 1993; Stewart and Kensler, 1986; Kensler and Woodhead, 1995), shadowed, freeze-fractured myosin filaments from frog skeletal muscle (Cantino and Squire, 1986), and myosin filaments from freeze-substituted frog skeletal muscle (Craig et al., 1992). These studies have shown the presence of the perturbation in 3-stranded crossbridge arrangement in all these species, consistent with the perturbation first described by Huxley and Brown (1967), but could not definitively demonstrate the nature of the perturbation. Myosin filaments in vertebrate skeletal muscles have also been studied in 3D (Stewart and Kensler, 1986; Eakins et al., 2002; AL-Khayat and Squire, 2006; AL-Khayat et al., 2006), but only recently has single particle analysis been used (AL-Khayat et al., 2004, 2006).

Myosin filament 3D structure has also been extensively studied in several well-ordered invertebrate striated muscle tissues such as insect flight muscle, tarantula, *Limulus*, scorpion and scallop (Crowther et al., 1985; Stewart et al., 1985; Morris et al., 1991; Vibert, 1992; Offer et al., 2000; AL-Khayat et al., 2003). In these cases helical reconstruction by the Fourier-Bessel approach has usually been applied, since the myosin head arrays in these muscles appear to be helical. The exception is the ground-breaking study by Woodhead et al. (2005) who applied single particle analysis and helical averaging to cryo-EM images of tarantula myosin filaments and achieved a very detailed 3D reconstruction. We have also applied the single particle approach to other helically ordered myosin filaments from both insect and scallop invertebrate striated muscles (AL-Khayat et al., 2008a, b).

Recent 3D-information about the structure of the vertebrate striated muscle myosin filament from fish skeletal muscle has been achieved both by modelling of the X-ray diffraction data (Hudson et al., 1997; AL-Khayat and Squire, 2006) and by single particle analysis of EM images (AL-Khayat et al., 2006). Both these studies have provided better structural detail than had been obtained previously (Eakins et al., 2002) and they started to quantify for the first time the perturbation of the crossbridge array in the fish myosin filament. This work, together with the earlier 3D reconstruction of isolated frog skeletal muscle myosin filaments (Stewart and Kensler, 1986), has provided significant information about the crossbridge arrangement in the vertebrate skeletal muscle myosin filament. However, less is known about the structure of myosin filaments in vertebrate heart muscles. Although 2D-analysis of isolated cardiac thick filaments has been performed (Kensler, 2002, 2005a, 2005b; Kensler and Harris, 2008), a 3D reconstruction of the structure of the cardiac muscle myosin filament has not been performed.

In the present work, the 3D structure of myosin filaments isolated from normal mammalian rabbit cardiac muscle has been studied. Using the technique of single particle analysis of non-helical filamentous systems (Paul et al., 2004; AL-Khayat et al., 2004; AL-Khayat et al., 2006) we produced a 3D reconstruction at about 40 Å resolution of the head arrangement in the C-zone region of relaxed rabbit cardiac myosin filaments. The results are very similar to the published structure of the vertebrate skeletal (fish) muscle myosin filament from EM 3D reconstruction (AL-Khayat et al., 2006) and from X-ray diffraction modelling (AL-Khayat and Squire, 2006), possibly suggesting a common structural theme for vertebrate striated muscle myosin filaments (cardiac and skeletal). However, for the first time, we show evidence that at low radius from the filament axis, the myosin heads origins are closer to being on a perfect helix than at high radius and that especially the azimuthal perturbation becomes more marked as the radius increases. This new structure serves as a starting point from which to understand the effects of the mutations in myosin and C-protein (MyBP-C) associated with different cardiomyopathies.

## 2 Materials and methods

### 2.1 Electron microscopy

Myosin filaments were isolated from rabbit ventricular muscle in relaxing solution as described by Kensler (2002, 2005a). The isolated myosin filaments were applied to a thin carbon film support on holey carbon grids and negatively stained. Only filaments lying over holes were used in the current analysis. Electron micrographs were collected under minimal dose conditions on a JEOL 1200 electron microscope at 40 K magnification.

### 2.2 Preparation of images for single particle analysis

**Fig. 1:**
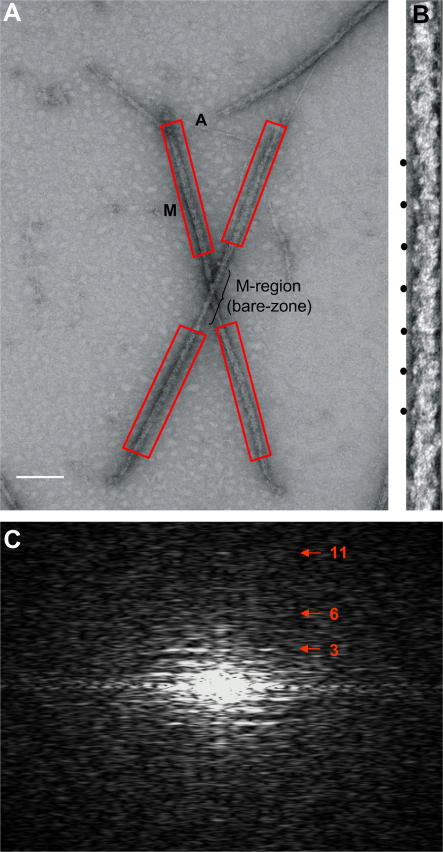
(A) Overview electron micrograph of isolated myosin filaments (M) from the ventricular muscle of normal rabbit heart in the relaxed state, viewed in negative stain over a hole in the support film. Some actin filaments (A) can be seen in the background. The helical arrays of myosin heads are evident in each half of the bipolar myosin filaments (scale bar 2000 Å). (B) Typical half-filament selected from the micrograph such as in (A), shown with the M-region (bare-zone) towards the bottom and showing the characteristic 430 Å axial repeat (black circles). (C) Calculated Fourier transforms of the half-filament shown in (B). Orders of the 430 Å repeat are shown in red numbers. The spacing of the sixth order of the 430 Å repeat, the 71.5 Å meridional reflection, was used to calibrate the magnification and to adjust the sampling of each half-filament from all the different micrographs to be exactly 7.54 Å/pixel. This sixth order meridional reflection was particularly strong in most of the Fourier transforms. The 11th order of the 430 Å repeat corresponding to 39 Å resolution (the titin sub-repeat) is also visible.

A total of 52 electron micrographs were digitised using a Nikon Super Coolscan 8000 16-bits per colour pixel scanner at a step size of 6.35 μm/pixel (equivalent to 1.58 Å sampling in the specimen). Digitised images for the whole micrographs were saved on the PC in TIFF format and were then transferred to a Linux UNIX operating system PC running the IMAGIC suite of programs (van Heel et al., 2000) to analyse them. Initially, they were converted to floating point IMAGIC format using EM2EM. The resulting images were then binned by a factor of 4 in both the *x* and *y* directions, resulting in a final step size of 6.35 Å/pixel and converted to MRC format for pre-processing using the MRC suite of programs (Crowther et al., 1996) and also using locally developed software. Regions were selected which contained intact half-filaments which were relatively straight, not overlapped by other actin and myosin filaments, and had readily identifiable bare-zones (Fig. 1(A)). Location of the bare-zone was essential to properly deduce the location of the C-protein stripes. The area selected on either side of each half-filament was also required to have as little background as possible (Fig. 1(B)), so as to reduce noise in the calculated Fourier transforms (Fig. 1(C)). Images of whole myosin filaments were cut into two halves with the whole bare-zone (M-band) included in each half-filament. In order to preserve polarity in the processing, half-filaments (i.e. from the M-band to the pointed end of the myosin filament) were then rotated to make each filament image vertical and oriented with its bare-zone (M-band) region at the bottom (Fig. 1(B)).

From the 52 available micrographs and using the above selection criteria, 153 half-filaments were identified. Half-filament images were floated in 2048 square arrays and their Fourier transforms computed (Fig. 1(C)). The sixth order of the 430 Å repeat, the 71.5 Å meridional reflection, which was strong in most computed Fourier transforms, was used to calibrate the magnification and to adjust the sampling of each half-filament from all the different micrographs to be exactly 7.54 Å/pixel. The majority of the Fourier transforms for the filaments showed up to the 11th order of the 430 Å repeat corresponding to 39 Å resolution (the titin sub-repeat; Fig. 1(C)). The correctly scaled half-filament images, in MRC format and with the pixel size accurately scaled to 7.54 Å/pixel, were then read again into IMAGIC and converted back to IMAGIC format using the EM2EM command.

All the further single particle image analysis was carried out within IMAGIC. The modified exact filter method for back-projection described in Paul et al. (2004) was used for calculating the 3D reconstruction. This allows the thickness of the central section to be adjusted taking into account the fact that the diameter of the filament is less than the size of the cube. 3D structures were visualised with both IMAGIC and PyMOL (DeLano, 2002).

## 3 Results

### 3.1 Selection of myosin filament segments

**Fig. 2:**
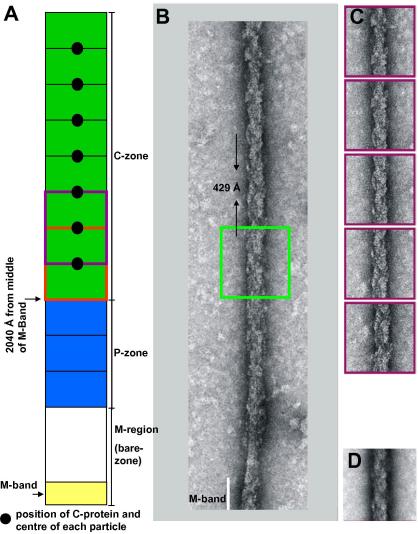
(A) Schematic diagram showing the different A-band regions within the myosin half-filament as defined by Sjostrom and Squire (1977a, 1977b) starting with the half M-band at the bottom, then the half bare-zone (M-region), the P-zone and the C-zone. Particles were selected from the C-zone only, each particle being just over 2 × 430 Å repeats long and with a C-protein stripe (maroon circle) in the middle. (B) Half-filament oriented with the M-region (bare-zone) at the bottom. The first particle was selected at the position shown by a green square of size 128 pixels, equivalent to just over 2 × 430 Å, and whose edge was at a distance 2040 Å from the middle of the M-band. (C) Some of the seven successive boxed segments each of length 128 pixels selected by stepping along the single half-filament in (A) at 430 Å intervals. (D) The average sum of the seven particles some of which are shown in (C).

Fig. 1(A) shows a typical micrograph of negatively stained isolated rabbit cardiac myosin filaments that contain good detail and from which half length myosin filaments were selected as shown in Fig. 1(B). As previously reported (Kensler, 2002, 2005a), well-preserved rabbit cardiac muscle myosin filaments, which are bipolar, have regular myosin head arrays in each half-filament with clear bare-zones (M-regions) halfway along. M-band protein density was sometimes visible in the middle of the M-region. The filament Fourier transforms showed meridional peaks out to the 11th order of 430 Å at 39 Å (Fig. 1(C)). Our aim in this study was to produce a 3D reconstruction of the structure of the myosin filament from only within the C-zone area (Sjostrom and Squire, 1977a, 1977b). This should result in a closer representation of the C-protein distribution in the final 3D structure than has been achieved before (AL-Khayat et al., 2006). Previously particles were selected from the whole of the half-filaments and thus included data from the P-zone and D-zone regions of the A-band as well as the C-zone (Fig. 2(A)).

### 3.2 Locating C-protein along the filaments

To locate the C-zone, 1D density profiles were calculated for each of the 153 individual half-filaments examined. These half-filaments ranged in length from 6000 to 7000 Å. Their 1D profiles were aligned together by cross-correlation using a program especially developed for this purpose, Cross-Corr (Knupp, C. unpublished). The Cross-Corr program was used to align and sum these 1D profiles to give averages in order to locate precisely the positions of C-protein. Seven stripes of higher density were observed. The first particle was taken to start at a distance 2040 Å from the middle of the M-band (Fig. 2(A)) and to include at its centre the first C-protein line, C3, reported by Sjostrom and Squire (1977a, 1977b) and Bennett et al. (1986; they called it C5). Beyond this, six further strong peaks were seen separated by approximately 430 Å. These are referred to as C6, C9, C12, C15, C18 and C21 as in Sjostrom and Squire (1977a, 1977b).

### 3.3 Particle selection, alignment, classification and 3D reconstruction

From a dataset of 153 half-filaments, 802 boxed square segments, each of length 128 pixels (equivalent to 7.54 Å/pixel × 128 pixels = 965 Å) containing just over 2 × 430 Å repeats (just over six 143 Å-myosin crown levels) were selected by stepping the box along the half-filaments at intervals of 430 Å (Fig. 2(A)). The chosen box length was used as a compromise between the easier alignment of a longer particle and a better compensation of the effects of any filament bending or distortion obtained with a shorter particle. Up to seven particles were selected from each half-filament to ensure that they only include the C-zone area.

**Fig. 3:**
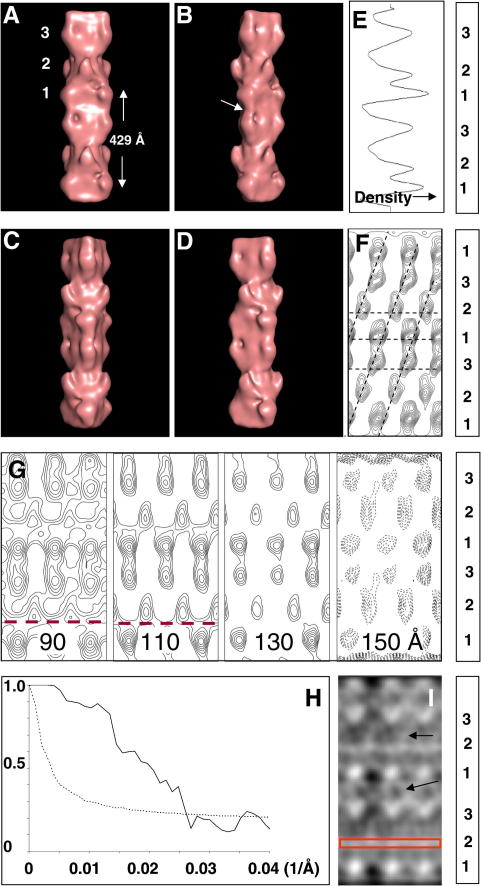
(A–D) Surface views of 3D reconstructions of the rabbit myosin filament obtained by the single-particle EM analysis and displayed using PyMOL (DeLano, 2002). The reconstruction is shown in four views at different angles around the filament long axis (in steps of 30°) to illustrate the different crown structures; (A) view with the myosin heads on the second crown facing the viewer and (C) view with the myosin heads on the first and third crowns facing the viewer. Note that the two views in (A) and (C) are quite distinct because of the different perturbations in the crowns. The arrow in (B) points to longitudinal density connecting the projected myosin head masses on levels 3 and 1. (E) 1D density profile of the maps shown in (A–D) illustrating the strong density, presumed to be C-protein, on crown 1. (F) Circumferential section at a radius of 110 Å from the filament axis in the fish EM reconstruction of AL-Khayat et al. (2006) shown as a contour plot. (G) Circumferential sections at radii of 90, 110, 130 and 150 Å from the filament axis in the current rabbit EM reconstruction (as in (A–D)), shown as contour plots. There is a weak circumferential density (dashed red lines) just above level 1. (H) Estimation of the resolution of the reconstruction using a Fourier Shell Correlation plot (solid line) obtained by comparing two independent reconstructions each representing half the dataset of the final 3D map compared with the half-bit curve (dashed line) according to the definitions of van Heel and Schatz (2005). The intercept between the two curves is at about 38.6 Å. (I) Circumferential section at a radius of 75 Å from the filament axis in the current rabbit EM reconstruction (as in (A–D)), shown here as a density. The red box points to density above crown 1 as in the dashed lines of G. The arrows point to a network of densities in the regions between the positions of the myosin heads masses on crowns 1 and 3 and 2 and 3 that could be attributable to myosin rod mass, C-protein and/or titin. All views in (A) to (G) and (I) are with the M-band towards the bottom and crown numbers labelled on the panels to the right.

Since the three crown levels within the 430 Å repeats were different, it was essential to make sure that the selected particles were in proper axial register. The aim was to align all the segments and classify them so that each class will be the same structure but rotated about the filament long axis. The simplifying assumption was made that in the main part of the bridge regions of vertebrate myosin filaments within the C-zone area, the 430 Å repeats are all the same. In order not to mix the different crowns within a 430 Å repeat, the alignment was checked thoroughly by calculating the 1D density profiles for the sum of the particles that came from each of the 153 individual half-filaments. A typical example of the sum of particles within a half-filament is shown in Fig. 2(D) and its corresponding 1D profile is similar to that shown in Fig. 3(E). The 1D density profiles for the sum of particles of each of the 153 half-filaments were then aligned together and the positions of the peaks calculated. The shift values needed to superimpose similar peaks were found for each half-filament. The sums of the shifted particles within each individual half-filament, together with their corresponding 1D profiles, were then re-calculated to check that they were all properly aligned.

A circular mask was applied to the dataset of 802 images and these were then rotationally and translationally aligned to an initial reference corresponding to the average sum of all the particles. Since the selected filament images were already quite accurately rotationally and translationally aligned, the angular range in the alignment was restricted to ±15° in order to preserve the polarity of the filaments. Also, the axial shift along the filament long axis was restricted to be between −50 Å and +50 Å to prevent the crown levels getting out of step. The aligned images were then classified by Multi-variate Statistical Analysis (MSA) (van Heel et al., 2000) to group those particles corresponding to the same view.

### 3.4 Angular assignment and class refinement

Because the aligned filament segments are likely to be related by rotation about a single axis (the filament long axis) with little out-of-plane rotation normal to this axis, *ab initio* angular assignment by standard angular reconstitution is not possible (Paul et al., 2004). Therefore the approach adopted here (as suggested by Paul et al. (2004)) was based on using a starting reference 3D model. The starting model was used to create an ‘anchor set’ of 2D re-projections as a reference from which to assign projection angles to class averages derived from the individual segments. By convention, these projection angles are defined by the three Euler angles *α*, *β* and *γ*. *α* is the rotation angle in the plane of the image (i.e. the plane of the EM grid), *β* is the out-of-plane tilt angle (to allow for the fact that the filaments may not lie perfectly flat on the grid or the grid itself may not be flat) and *γ* is the rotation angle around the filament long axis. We had already developed and implemented the same procedure in the previous study on the fish skeletal myosin filaments (AL-Khayat et al., 2006). In that work a number of strategies were used to test the uniqueness of the final 3D reconstruction. These included using various different starting 3D models to generate the anchor sets so as to avoid any model bias, using a totally independent angular assignment method where no starting model was required (Patwardhan et al., 2004), and using different software, EMAN (Ludtke et al., 1999). All these different methods led to essentially the same final 3D reconstruction, giving us confidence in the methodology that we were using. In the present study, we therefore used a single first reference 3D model to generate an anchor set of 2D projections to use for angle assignment (Euler angles) for each of the class averages. This reference model was generated by calculating a 3D reconstruction of a single class average with Euler angles *α*, *β* and *γ* of 0°, 90°, and 0°, respectively, assigned to it as its angles of view and imposing 3-fold symmetry around the long axis. The anchor set produced from this reference 3D map was used to provide a first set of Euler angles for all the class averages. An improved 3D reconstruction could then be calculated. 2D re-projections of this new 3D map were used to make a new set of reference images with which to re-align the original raw image dataset of the 802 particles using Multi-Reference Alignment (MRA), to re-classify them and to carry out a further process of angular reconstitution/assignment. This procedure was repeated with the class images gradually improving, and refinement was stopped when no further significant change was observed and the assigned angles became stable.

### 3.5 The final 3D reconstruction

The final 3D reconstruction (Fig. 3(A–D); M-band direction downwards) was calculated with imposed C3 symmetry using the best 17 class averages containing a total of 301 particles and using the modified weighted back-projection method of Paul et al. (2004). According to the Fourier Shell Correlation (Fig. 3(H)), the resolution of the reconstruction lies between 40 Å (0.5 bit criterion, van Heel and Schatz, 2005) and 48 Å (0.5 correlation coefficient criterion), and the 3D image was filtered to include frequencies from 300 Å up to 40 Å resolution. The contour threshold used to show these images corresponded to the theoretical volume of the myosin molecules within the length represented (9 whole molecules/430 Å repeat) plus an additional 15% to account for non-myosin proteins (titin and C-protein) present in the myosin filament. The reconstruction shows slightly more than two full 430 Å repeats of the myosin filament and the two successive 430 Å repeats are substantially similar to each other. Although these regions contain some overlapping image data, they were not averaged together during the analysis and the structural similarity of the successive 430 Å repeats can be taken as support for the validity of the reconstruction.

Four views related by a 30° rotation around the filament axis are included in Fig. 3(A–D) to illustrate the different crown structures as well as their angular perturbation from helical symmetry. Surface density features in the 3D map, presumably mainly myosin heads, can be seen following a perturbed helical path. There are three levels of myosin heads within each 430 Å repeat, but they are not identical. On levels labelled 1 and 3, the projecting masses appear to have approximately the same azimuthal positions, whereas those on levels labelled 2 are at quite a different azimuth. The overall appearance suggests that there is on average a large departure from helicity in the azimuthal positions of the crown densities from the constant 40° angular rotation expected from ideal helical symmetry.

The azimuthal and axial perturbations from helical symmetry are further demonstrated in Fig. 3(G) which shows the density in circumferential sections (like a radial net) through the 3D reconstruction at radii of 90, 110, 130 and 150 Å from the filament axis. For comparison, the circumferential section at a radius of 110 Å from the filament axis in the EM reconstruction of fish skeletal muscle myosin filaments (AL-Khayat et al., 2006) is shown as Fig. 3(F) where the same leftwards shift of mass on level 1 is seen.

**Fig. 4:**
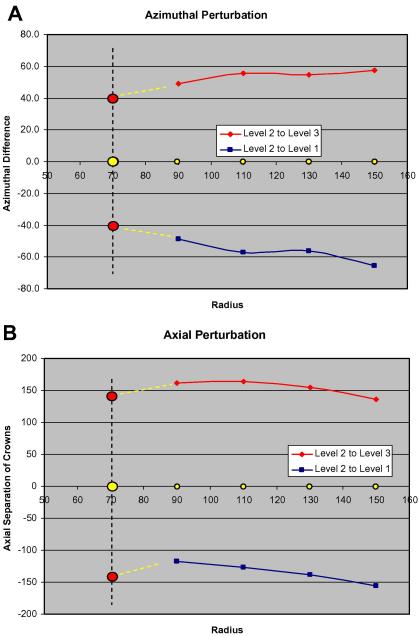
The azimuthal (A) and axial (B) perturbations measured relative to level 2 and plotted as a function of radius from the filament axis. The red circles show the expected start positions if the myosin head origins are on a perfect helix. The yellow circles show the crown 2 level used as a comparison (i.e. zero difference from itself). The yellow dashed lines shows the way in which the perturbations between levels 2 and 1 and 2 and 3 would need to change for the head origins to be on a perfect helix. In (A) the azimuthal perturbation appears to be tracking back towards the 40° for a perfect helix. In (B) the level 2 to level 3 axial difference starts around 140 Å at high radius, increases away from this as the radius reduces, but then appears to be coming back towards the perfect helix value (143.4 Å) at lower radius. On the other hand the axial perturbation between levels 2 and 1 appears to be consistently reducing towards lower radii. Further low radius data at higher resolution will be required to see if this perturbation also returns to 143.4 Å.

Analysis of the positions of centres of mass on each crown in Fig. 3(G) reveals an interesting feature. Fig. 4(A and B) show how the azimuthal and axial perturbations vary with radius. The azimuthal and axial displacements of density peaks on crowns 1 and 3 relative to those on crown 2 are plotted as a function of radius between 90 and 150 Å. Fig. 4(A) shows that there is a very clear trend for the angular separation of peaks on crowns 1 and 3 relative to crown 2 to tend towards the 40° expected for a perfect helix as the radius reduces. In Fig. 4(B) for the axial perturbations it is also conceivable that the axial shift is tending towards 143 Å at low radius between levels 2 and 3 as indicated by the extrapolated yellow lines. However, what happens between levels 1 and 2 is less clear cut.

In the present reconstruction, as in the fish muscle myosin filament (Fig. 3(F)), additional density which may be C-protein is associated with crown 1. However, there also appears to be extra density running from crown 1 down to the adjacent crown 3 (Fig. 3(G)) which could also correspond to C-protein, titin, or density associated with the myosin rods in the backbone.

## 4 Discussion

### 4.1 Single particle analysis of vertebrate mammalian cardiac myosin filament structure

The technique of single particle analysis of non-helical filamentous systems involving the creation of particles by dividing the myosin filaments into segments has already been applied to produce a 3D reconstruction of the myosin head arrangement in relaxed vertebrate striated (fish) skeletal muscle (AL-Khayat et al., 2004, 2006). These filaments deviate from ideal helical symmetry and their structure can not be accurately determined by traditional Fourier-based helical 3D reconstruction. Our new approach has allowed the 3D analysis of EM images of these non-helical filaments without invoking helical symmetry (AL-Khayat et al., 2004, 2006). The method can preserve perturbations in the myosin head array within the 430 Å repeat length which are otherwise averaged out in helical reconstructions.

The new approach was originally applied to the 3D structure of myosin filaments in vertebrate skeletal muscle, but until the present work it had not been applied to the medically important 3D structure of myosin filaments in vertebrate heart muscles. The current analysis not only had the benefit of extending the work from vertebrate skeletal to cardiac myosin filaments, but was also an improvement in that the analysis was carried out using only the C-zone part of the filament. Studying cardiac myosin filament structure in the normal state is an essential starting point from which to understand the mechanisms of the diseased system, in particular the effects of the mutations in myosin and its accessory proteins such as C-protein (MyBP-C) associated with different cardiomyopathies.

**Fig. 5:**
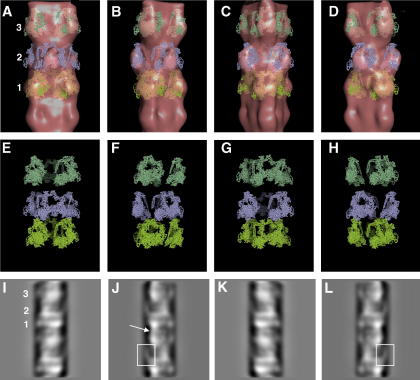
(A–D) The best fit of Wendt et al. (2001) into the current EM map viewed at four different angles around the filaments long axis (in steps of 30°) as in Fig. 3(A–D) displayed using PyMOL (DeLano, 2002). (E–H) The fitted Wendt structure shown without the 3D map to reveal the detailed differences in head orientations on each crown. (I–L) 2D projections of the 3D map projected at the same angle of views shown in Fig. 3(A–D). The levels are numbered in the same way as in Fig. 3. See text for arrows and boxes.

Good evidence that our current 3D map is a reliable structure is that it helps to explain the features observed in the myosin filament images determined by 2D filtration by Kensler (2005a) using the same images that were used in our current single particle analysis. Fig. 5(I–L) shows projected views of our 3D map which reproduce the features seen in the filament images of Kensler (2005a). In particular what was described as the “comma-shaped” heads on level 2 (boxed density in Fig. 3(B) in Kensler, 2005a) is now seen as a projecting density which is curved upwards in the 3D map (Fig. 3(A–D)). The comma-shape feature is also boxed in Fig. 5(J and L)). The projected views of the 3D map are also consistent with the “saw-tooth” pattern of myosin head densities described by Kensler (2005a) (labelled by red lines in Fig. 4(B) in Kensler, 2005a) and clearly demonstrated in the 2D re-projection of the current 3D map (Fig. 5(I–L)). It is now evident that this “saw-tooth” pattern observed in both fish skeletal and rabbit cardiac myosin filaments is due to both the 3-stranded arrangement of the heads as well as the azimuthal perturbation inherent in both filaments.

### 4.2 Axial and azimuthal crown perturbations

Comparison of our new map for rabbit cardiac muscle myosin filaments with that for fish skeletal muscle myosin filaments (AL-Khayat et al. 2006; Fig. 4) shows that the angular appearance of the single particle 3D reconstructions and the clear axial and azimuthal perturbations are common to both filament types. In both cases levels 1 and 3 have heads pointing on average in roughly the same azimuthal directions compared with level 2 which in both cases is rotated by roughly 60° compare Fig. 3(F) with Fig. 3(G; 110 Å). The radial perturbation is minor in both the fish and rabbit myosin filaments. Comparison between the angles and axial spacings/separations between the three levels in both fish skeletal and the current rabbit heart myosin filaments are shown in Table 1. It can be seen that the angular perturbation in rabbit is slightly larger than in fish. It can also be seen that the angular separations are closer to 60° in rabbit compared to the corresponding ones in fish. This could be due to the current C-zone analysis allowing better resolution of the angles than those in fish. Moreover, the axial spacing between levels 2 and 1 is lower in rabbit (−134.4 Å) compared to that in fish (−149.0 Å) whereas the spacing is larger between levels 2 and 3 in rabbit (154.0 Å) compared to fish (135.0 Å).

The axial perturbations measured for rabbit cardiac myosin filaments by Kensler (2005a) between what we define as levels 2 and 1, and 2 and 3 were −132.9 Å and 153.0 Å, respectively, as measured from six filtered images. [Note that Fig. 4(E) in Kensler (2005a) referred to our levels 1, 2 and 3 as 2, 3 and 1, respectively. His level 0 in Fig. 4(E) (Kensler, 2005a) is our level 2.] Our new values based on the average of data plotted in Fig. 4 are very similar at −134.4 Å and 154.0 Å, respectively (Table 1).

### 4.3 Location of non-myosin protein densities

There is evidence from the earlier work on fish skeletal myosin filaments that the main C- (or X-) protein density is located at level 1. A similar conclusion can be reached for the rabbit cardiac myosin filaments (Fig. 3(E)) where the strongest projected density is also at level 1. A further important observation in AL-Khayat et al. (2006) was that the azimuthal perturbation between the three crowns probably adjusts the head locations to fit perfectly within the hexagonal filament lattice common to all vertebrate striated muscles so that the myosin heads do not clash with neighbouring actin filaments. The same conclusion can be reached for the cardiac myosin filaments studied here. That the myosin filaments from different species and from skeletal and cardiac muscles show similar perturbations and a similar location for C- (X-) protein argues that there may be a single basic three-dimensional plan common to all vertebrate striated muscle myosin filaments, presumably with minor variations.

A feature seen in the fish myosin filament reconstruction is that there is axial density linking levels 3 and 1 (see Fig. 3(F)). Similar density is absent between levels 1 and 2 and 2 and 3. However, this linking density between levels 3 and 1 is rather more marked in the cardiac reconstruction (arrowed in Fig. 3(B), Fig. 3(G; 110 Å), arrowed in Fig. 5(J)) which could be due to the selection of particles in this case from only the C-zone. We therefore tentatively attribute this “extra” density to C-protein in the cardiac thick filament and infer that similar connecting C-protein density also occurs in fish skeletal muscle myosin filaments. In the X-ray diffraction modelling (see Fig. 5(b) and (d) in AL-Khayat and Squire, 2006) a similar region of longitudinal connecting density between levels 3 and 1 was also present. Since C-protein and other accessory proteins were not included in the analysis, the best fit model had the myosin heads on level 1 dipping down and almost touching the heads on level 3 to account for this connecting density. The current 3D-EM reconstruction suggests the alternative possibility that this axial connecting density may be due to the presence of C-protein at this location.

Skeletal muscle C-protein is known to be a modular structure composed of ten fibronectin 3-like (Fn-3) and immunoglobulin I-like (Ig-I) domains (termed C1–C10), with an N-terminal Pro-Ala-rich domain; (Pro-Ala)-C1(IgI)-C2(IgI)-C3(IgI)-C4(IgI)-C5(IgI)-C6(Fn3)-C7(Fn3)-C8(IgI)-C9(Fn3)-C10(Ig) (Offer et al., 1973; Bennett et al., 1986; Vaughan et al., 1993). In the case of cardiac C-protein there is an additional N-terminal domain C0(Ig) before the Pro-Ala domain and there is an insertion between the first two Ig domains (C1 and C2) (Yasuda et al., 1995). The connectivity of mass in our reconstruction running between the projected myosin head masses of levels 1 and 3 (arrowed in Figs. 3(B) and 5(J)) could be titin or C-protein. If the density corresponds to C-protein, this may be consistent with the C-terminal part of the C-protein (domains C6 to C10) running axially along the axis of the myosin filament as proposed by Squire et al. (2003). It could be this longitudinal connecting density that causes the myosin head masses on levels 3 and 1 to be kept azimuthally aligned. The C0 to C5 part of C-protein is thought to be able to project out from the myosin filament and to bind to actin (Squire et al., 2003).

A weak feature of uncertain origin in our new map and seen in the circumferential sections of the current 3D reconstruction at low radius (e.g. dashed red lines in Fig. 3(G; 90 and 110 Å radius), is a continuous circumferential density halfway between levels 1 and 2. Although this feature was less continuous in the 3D map of fish skeletal muscle (Fig. 3(F)), a similar circumferential band of density at a radius of 50 Å was present in the earlier 3D map of the isolated frog skeletal muscle thick filament (Stewart and Kensler, 1986; Fig. (11a); note that the numbering of the crossbridge levels in the frog myosin filament map is different from the current rabbit myosin filament map). It could be argued that this continuous density in the circumferential section of the rabbit cardiac myosin supports the model for the structure of C-protein proposed by Moolman-Smook et al. (2002) with a trimeric collar arrangement around the myosin filament. At present it is unclear whether the difference in the density between the rabbit cardiac and fish skeletal myosin maps is due to a genuine difference between the filaments or is a difference due to the present more selective analysis where only the C-zone area was included.

We have further investigated the locations of non-myosin densities by investigating the circumferential sections at a radius closer to the backbone of the filament where the myosin heads do not contribute as much and therefore any densities seen may be attributed to myosin rods, C-protein or titin. This is shown in Fig. 3(I) which is a circumferential section at a radius of 75 Å from the filament axis in the current rabbit EM reconstruction. The red box in Fig. 3(I) highlights the circumferential density above crown 1, discussed earlier (Fig. 3(G; 90 and 110 Å dashed red lines)) that may conceivably be attributed to a C-protein collar. In addition to this there is a weak meshwork of density, possibly attributable to titin or other features of the filament backbone, in the regions between what are taken to be the myosin head masses on crowns 1 and 3 and 2 and 3 as shown arrowed in Fig. 3(I). These densities were not seen in the reconstruction of the fish skeletal myosin filaments of AL-Khayat et al. (2006) and may correlate with the strong meridional reflections on the 10th and 11th layer lines in the transforms of the rabbit cardiac myosin filaments (Kensler, 2005a, 2005b). The reflection on the 11th layer line corresponds to the 39 Å spacing expected for the domains of titin. This weak meshwork of density may correspond to the pattern of beaded striations at this spacing on the backbone reported by Kensler (2005b).

### 4.4 The arrangement of myosin heads in cardiac muscle myosin filaments

We have investigated the possible organisation of the myosin head pairs in our new 3D map by asking whether the observed densities are consistent with the 2D crystal structure of vertebrate smooth muscle two-headed myosin determined by Wendt et al. (2001). In order to obtain the best fit and due to the fact that the crowns were not identical, the two-headed Wendt structure was modelled in different orientations in each crown (Fig. 5(A–H). The fitting was done for each crown individually within PyMOL by orienting the Wendt structure by hand within one of the three projecting mass densities on that crown. The other two 3-fold-symmetry-related Wendt structures on each crown were then positioned using the 3-fold transformation command in the CCP4 software package (http://www.ccp4.ac.uk/). It appears that crowns 1 and 3 can both accommodate the Wendt model quite well, but that crown 2 does not fit. This agrees with the conclusions for fish skeletal myosin filament (AL-Khayat et al., 2006) in that the Wendt structure fitted reasonably well into two crowns (levels 1 and 3) but not the third (level 2). We conclude that the Wendt et al. (2001) structure is not a totally universal myosin head arrangement even though it fits well to the helically averaged crowns in tarantula invertebrate striated muscle myosin filaments (Woodhead et al., 2005). Instead it appears to occur on two of the three levels of fish skeletal and rabbit cardiac muscle myosin filaments, but not on the third.

Moreover, the Wendt model fits much better within crown 1 in the current rabbit cardiac myosin filament map than it did on crown 1 in the fish skeletal myosin. In addition, in the current reconstruction, the Wendt model fits somewhat less well within crown 3 than it did on the same crown in fish skeletal myosin. The projected myosin head density on level 2 in the current 3D map is more curved upwards (Fig. 3(A–D), Fig. 5(A–D)) than observed on the same level in the fish skeletal myosin filament (AL-Khayat et al., 2006). The difference in the fitting between the current map and the previous fish skeletal could be due to the fact that myosin filaments are different in cardiac and skeletal muscles. Obtaining a map at higher resolution would be beneficial in order to assess this difference in more detail.

There could be several possible reasons for the difference observed in the fit of Wendt model to the third level of fish skeletal and rabbit cardiac myosin filament compared to the very good fit in tarantula myosin filaments (Woodhead et al., 2005). One of these possibilities could be that these four species have different regulatory systems in that that both vertebrate smooth (Wendt model) and tarantula striated muscles are myosin-regulated by phosphorylation of their regulatory light chains (Aksoy et al., 1976; Sobieszek, 1977; Craig et al., 1987), whereas both fish skeletal and rabbit cardiac muscles are regulated by calcium binding to their thin filaments (Kendrick-Jones et al., 1970; Lehman and Szent-Gyorgyi, 1975).

Recent work by Jung and Craig (2007) reported preliminary results that show that myosin molecules isolated from tarantula and *Limulus* striated muscles (both phosphorylation-regulated) can have similar head–head interactions to those seen in vertebrate smooth and scallop striated myosin. Unlike these myosin-regulated muscles, head–head interaction was not observed in vertebrate skeletal and cardiac muscle myosins, in agreement with previous shadowing observations. However, their preliminary data showed that these unregulated myosin molecules can also exhibit head–head interactions if blebbistatin is bound to the heads (slowing phosphate release by promoting the ‘closed’ conformation of switch 2 in the motor domain of the head). Therefore the Jung and Craig (2007) results support the idea that myosin head–head interaction may be quite a common motif in the inhibited state of myosin II molecules. Nevertheless, vertebrate and invertebrate systems are evolutionarily widely separated. In addition to the fact that the various species have different regulation systems in that smooth, tarantula, *Limulus* and scallop myosins are all myosin-regulated whereas both skeletal and cardiac are thin filament-regulated, the tarantula, *Limulus* and scallop myosin filaments are all perfect/true helical systems. This true helical symmetry requires that there are the same orientations, configurations and interactions between the two-heads on each crown. On the other hand, vertebrate myosin filaments (skeletal and cardiac) are not true helical systems. The perturbation from helical symmetry could be reflecting differences in the configurations, interactions and functions of the two-headed myosin molecules on different crowns. It is therefore probably not surprising, as we have shown in the current rabbit cardiac myosin and the previous fish skeletal myosin (AL-Khayat et al., 2006), that the pairs of heads at the three crowns do not all share exactly the same arrangement (the Wendt model). The third possibility, along with the differences in regulation and helicity, is that tarantula, *Limulus* and scallop myosin filaments all have different compositions of accessory proteins (paramyosin core, etc.) compared to fish skeletal and rabbit cardiac (which have surface-located titin and C-protein). It could be that the extra surface proteins in vertebrate myosin filaments disrupt the fitting of the Wendt structure in one of the three levels, although it still fits well into the other two crowns. Fourthly, the differences in lattice arrangements between these species and the need to fit the underlying 9-fold structure of vertebrate myosin filaments into the 6-fold filament array, presumably requires there to be systematic differences in the head arrangements on different crowns.

We therefore conclude that, even if the vertebrate myosin heads do sometimes tend to take up the Wendt configuration in isolation, as per the work of Jung and Craig (2007) on isolated myosin molecules, the vertebrate myosin filaments with their perturbed helical myosin head arrangement, the presence of surface accessory proteins, the special lattice organisation in vertebrates, and their different regulatory system may all reflect the need for heads to depart from this arrangement on one crown.

### 4.5 The origin of the perturbations in vertebrate striated muscle myosin filaments

A method to test the effect of C-protein on the myosin filament structure is to study a system which does not have C-protein. Harris et al. (2002) reported the development of a knockout mouse for cardiac C-protein which shows hypertrophy and significant contractile defects, but retains normal sarcomeric structure. In a recent study, Kensler and Harris (2008) examined the structure of myosin filaments isolated from the C-protein knockout mouse heart muscle. They reported that myosin filaments isolated from the knockout mouse hearts appeared similar in length and diameter to the wildtype filaments, but the “forbidden” meridional reflections, thought to derive from a perturbation from helical symmetry in the wildtype filament, were weaker or absent in the Fourier transforms of the cMyBP-C^−/−^ myocardial myosin filaments. In addition, the crossbridge array in the absence of cMyBP-C appeared to be more easily disordered and sensitive to the negative staining conditions. These studies provide support for the idea that C-protein interactions with myosin may affect the myosin head arrangement as proposed by AL-Khayat et al. (2006). As noted by Kensler and Harris (2008), 2D studies of the filaments do not allow a determination of whether an azimuthal perturbation is present in the filaments. The large azimuthal perturbation observed for cardiac myosin filaments in this study would not give rise to the observed forbidden meridional reflections which arise from an axial crossbridge perturbation and/or the presence of additional mass on a 430 Å repeat. It is only 3D analysis, as in the present study, that allows a detailed and separate study of the axial and azimuthal components. This highlights the advantage of the single particle method in showing the azimuthal perturbation which is not evident from the observed forbidden meridionals in X-ray diffraction patterns and FFTs from 2D electron micrograph images.

The 3D reconstructions of the vertebrate thick filament (both the current rabbit cardiac and the previously published fish skeletal myosin filaments (AL-Khayat et al., 2006)) have clearly shown the presence of a perturbation in the crossbridge arrangement, but until now have not addressed the question of whether the perturbation in the crossbridges extends to the packing of the myosin rods in the backbone. We have shown here that the rabbit cardiac 3D map contained perturbations that were pronounced at large radius and reducing at smaller radius. We also note that our current rabbit cardiac myosin filament map complements the reported abstract of the wildtype mouse cardiac myosin filament of Perez-Zoghbi et al. (2007) who have stated that the three crowns in their map were not identical. However, our evidence suggesting the greater observed helical perturbation of the heads at higher radius compared to at lower radius from the filament axis is distinct to our current study. Future higher resolution 3D analysis work in both wildtype and C-protein knockout mouse heart muscle myosin filaments, as well as in our current studies of the isolated rabbit cardiac myosin filament, should help to select between the alternatives: EITHER that the azimuthal and axial perturbations within the myosin filament are an inherent feature of the packing of the myosin rods in a non-helical way within the filament backbone, OR that the myosin head origins lie on a perfect helix and that the conformational perturbations are confined to the myosin heads only and are due to the presence of titin, which does not have a 143 Å repeat, and the effect of titin in locating C-protein on every third crown level. Our evidence so far from the present analysis is that the second of these options is more likely.

## Figures and Tables

**Table 1:** Comparison of the angles and axial spacings/separations between the three levels in fish skeletal and the rabbit heart myosin filaments

	Angle between crown 2 and crown 1	Angle between crown 2 and crown 3
Fish	−53.96°	49.86°
Rabbit (this work)	−56.80°	54.30°
		
	Axial spacing crown 2 and crown 1	Axial spacing crown 2 and crown 3
Fish	−149.0 Å	135.0 Å
Rabbit (this work)	−134.4 Å	154.0 Å
Rabbit (Kensler, 2005a)	−132.9 Å	153.0 Å

The rabbit values in the current work are the averages of the values plotted in Fig. 4, whereas those for fish (AL-Khayat et al., 2006) are based on single projections.
